# Gram-Negative Bacteremia With Atypical Neurologic Presentation Following Tattoo Application

**DOI:** 10.7759/cureus.82373

**Published:** 2025-04-16

**Authors:** Adedolapo O Ojo, Desiree Marquez-Santos, Radmehr Rahemipour, Aniqa Aftabi, Alberto Meléndez-Garcia, Saira Shahab, Richard Steward

**Affiliations:** 1 Family Medicine, Episcopal Health Services, New York, USA; 2 Internal Medicine, Episcopal Health Services, New York, USA; 3 Infectious Disease, Episcopal Health Services, New York, USA

**Keywords:** atypical presentation, gram-negative bacteremia, neurologic symptoms, tattoo application, tattoo infections

## Abstract

Tattoo-related infections are typically caused by gram-positive bacteria like *Staphylococcus aureus* and present with localized symptoms. However, this case describes a 48-year-old diabetic woman who developed *Escherichia coli* bacteremia without any visible signs of infection at the tattoo site. She presented with systemic symptoms including fever, chills, and weakness, initially raising concerns for a neurological event. Laboratory tests confirmed gram-negative bacteremia, and the tattoo was identified as the likely source. The absence of local manifestations and the rare involvement of *E. coli* highlight the need for clinicians to consider systemic infections as a potential complication of tattooing, especially in high-risk patients. Early recognition and appropriate antibiotic therapy resulted in the patient’s full recovery. This case underscores the importance of infection control during tattooing and vigilance for atypical presentations.

## Introduction

Tattooing has become a growing trend worldwide, with approximately more than 35% of adults in the United States reported to have a tattoo [1]. In general, proper sanitary precautions are taken, and infections remain rare. The main risk factors for tattoo-related infections include poor hygiene and being a high-risk patient; being high-risk involves having an immunocompromised immune system, of note due to uncontrolled diabetes or immunosuppression such as from human immunodeficiency virus/acquired immunodeficiency syndrome [2,3]. Another source of tattoo-induced infection is contaminated ink. The tattoo ink can become contaminated from various sources, such as surfaces in the tattoo studio or poorly sterilized equipment or might even be intrinsically contaminated during production. It is common to have contamination rates of more than 10%. Two common bacteria found in contaminated inks are *Pseudomonas aeruginosa *and *Staphylococcus spp*. [4].

Studies report that 0.5-6% of individuals experience infectious complications after getting a tattoo [4]. Moreover, a study from 2024 has shown that the incidence of tattoo-related infections has increased since 2000 [2]. Tattoo needles puncture the epidermis, reaching the dermis and encountering nearby blood and lymphatic vessels. Most infections remain localized to the tattoo site, though some may progress to systemic infections [5]. However, a purely systemic infection with no local manifestation is extremely rare [5].

Two of the most common microbial agents responsible for tattoo-related infections are non-tuberculous *Mycobacteria* (NTM) and *Staphylococcus aureus* [2]. However, in some cases, other organisms, including gram-negative bacteria, have also been implicated [6]. Local infections can present as impetigo and, less commonly, as cellulitis or erysipelas [4]. As bacteria replicate, immune cells such as neutrophils and macrophages are recruited to the tattoo site. However, bacteria have virulence factors that can confound the immune system [7]. For example, *S. aureus* can form a biofilm that acts as a protective barrier, making it less accessible to immune responses such as phagocytosis by neutrophils and macrophages [8]. Also, NTM have the ability to evade macrophage lysosomal destruction [9]. If the local infection is not controlled, it can lead to bacteremia and manifest with constitutional symptoms such as malaise, weakness, fever, and chills. Eventually, the infection may progress to septic shock and multi-organ failure [4].

In this case report, we present a rare instance of tattoo-induced bacteremia caused by *Escherichia coli*. This case is unique because it lacked any local infection at the tattoo site and, in addition to typical constitutional symptoms, also presented with dysarthria and mild horizontal nystagmus.

This report aims to raise clinicians' awareness of atypical presentations of tattoo-related bacteremia, particularly those caused by uncommon gram-negative organisms such as *E. coli*. Early recognition of these unusual presentations is critical for initiating timely and appropriate empiric antibiotic treatment.

## Case presentation

A 48-year-old woman presented to the hospital with acute-onset symptoms, including dysarthria, generalized weakness, myalgias, nausea, lightheadedness, and chills. She had a known history of diabetes and had previously experienced similar symptoms attributed to hyperglycemia. The patient reported multiple recent exposures to individuals with COVID-19 but denied any history of stroke, smoking, coagulopathy, oral contraceptive pill use, prior deep vein thrombosis, or pulmonary embolism. Notably, she had a tattoo placed on her left shoulder a few days prior to hospitalization.

On physical examination, the patient was noted to have obesity, mild horizontal nystagmus, and a recently acquired tattoo on the left shoulder, which showed no signs of infection. The rest of the examination was unremarkable. 

**Figure 1:**
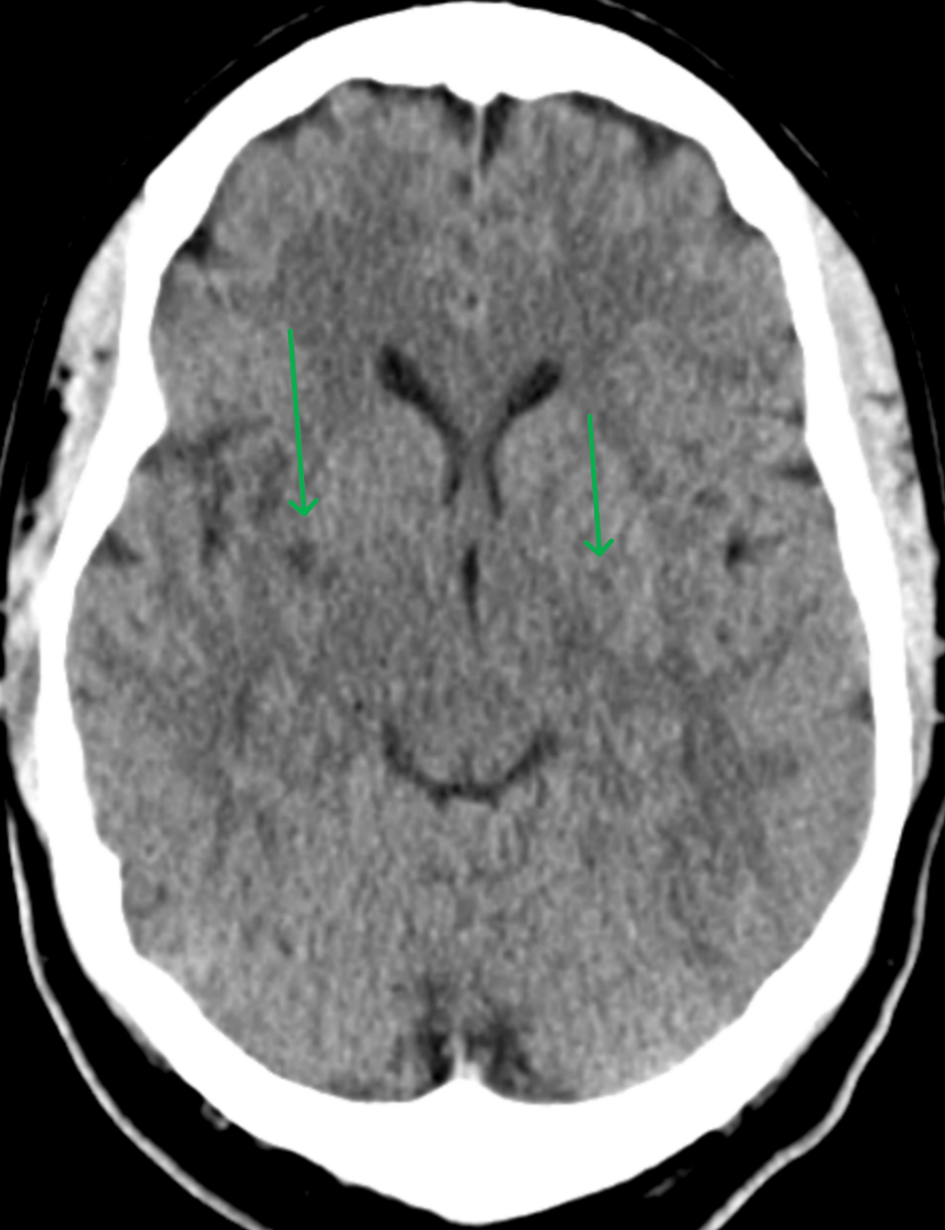
Cranial CT scan 1 Axial cranial CT scan showing lacunar infarcts in the bilateral basal ganglia and external capsule. No evidence of intracranial hemorrhage. The age of infarcts is indeterminate.

**Figure 2:**
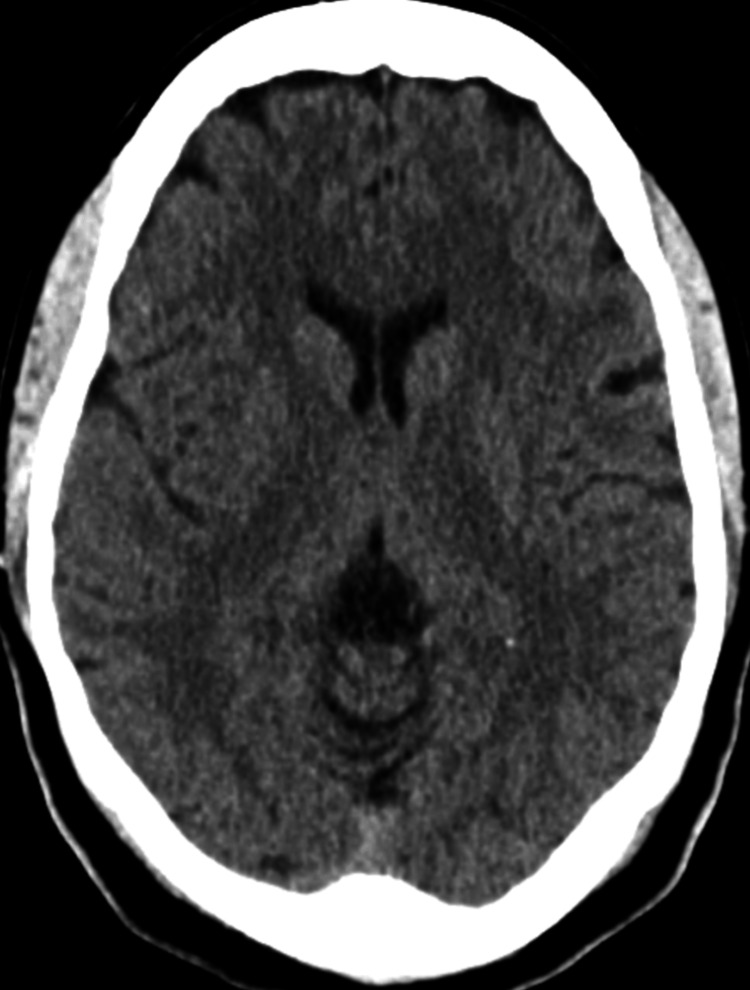
Cranial CT scan 2 Additional axial cranial CT image of the basal ganglia demonstrating no signs of intracranial hemorrhage.

Given her transient dysarthria, there was concern for a cerebrovascular accident (CVA); therefore, a cranial CT (Figures 1, 2) was obtained, which revealed lacunar infarcts in the bilateral basal ganglia and external capsules, but no intracranial hemorrhage.

**Figure 3:**
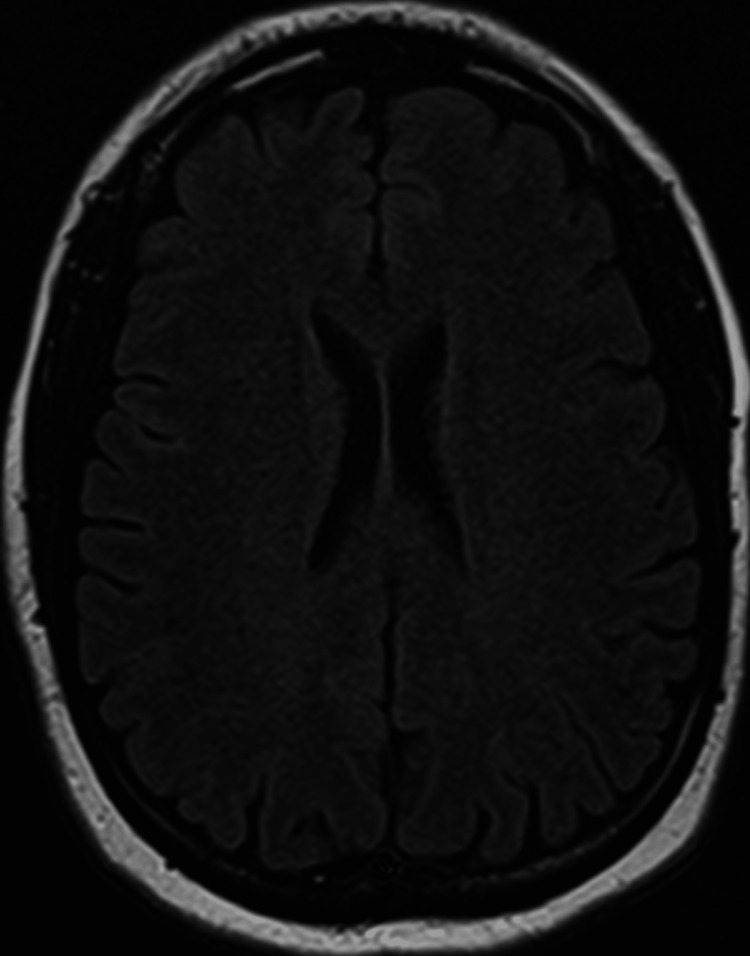
Brain MRI Axial Brain MRI showing no evidence for acute infarct in the basal ganglia. No mass or mass effect. No subdural hematoma.

A follow-up brain MRI (Figure 3) was negative for acute or chronic infarcts on diffusion-weighted imaging or fluid-attenuated inversion recovery sequences.

From a neurological standpoint, the findings on cranial CT were thought to be secondary to dilated perivascular spaces rather than chronic infarcts. Thus, a CVA was ruled out. The patient’s weakness and symptoms gradually improved.

Her vital signs were significant for a fever with a maximum temperature of 101.2°F and tachycardia, with a heart rate in the 110s bpm. Laboratory testing revealed neutrophilia (93.4%), hyponatremia (133 mmol/L), and hyperglycemia (237 mg/dL). Additional tests showed a positive hepatitis B virus IgG but were negative for hepatitis B surface antigen (HBsAg), hepatitis C antibody, influenza A and B, respiratory syncytial virus, and SARS-CoV-2 RNA. Urinalysis was positive for glucosuria (2+) and trace leukocyte esterase but negative for nitrites, and there were no symptoms of a urinary tract infection (UTI).

Given the recent tattoo and potential breach in skin integrity, there was concern for a bloodstream infection. Empiric antibiotic therapy was initiated on hospital day 3 with a single dose each of intravenous vancomycin (1 g) and ampicillin-sulbactam (Unasyn, 3 g). Importantly, blood and urine cultures had been obtained on hospital day 2, prior to antibiotic initiation, and both subsequently grew *E. coli*. In light of persistent fevers and confirmed gram-negative bacteremia, the Infectious Disease team recommended switching antibiotics. The patient was started on intravenous piperacillin-tazobactam (Zosyn) at a dose of 3.375 g every six hours, administered from hospital day 3 through day 7. This antibiotic choice was supported by susceptibility results. The patient remained febrile through hospital day 4 but subsequently defervesced with clinical improvement.

**Figure 4:**
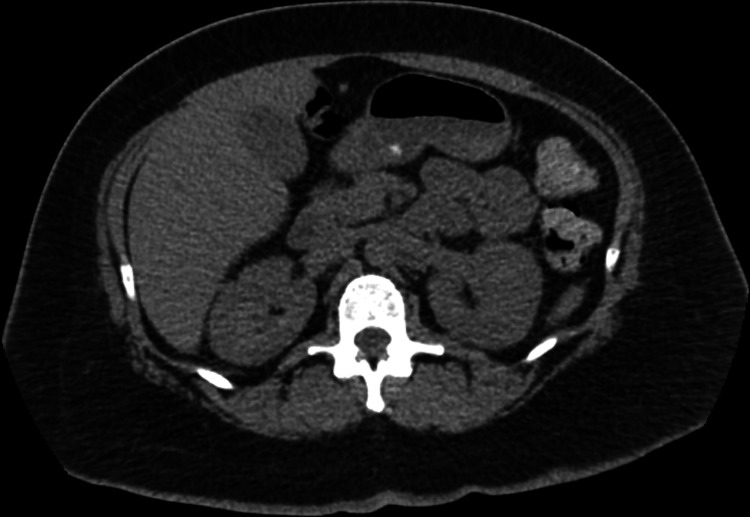
Abdomino-pelvic CT scan 1 Axial abdominopelvic CT scan at the level of the lung bases. The scan shows no evidence of bowel obstruction, perforation, or abscess. The appendix is intact, and no abnormalities are noted.

**Figure 5:**
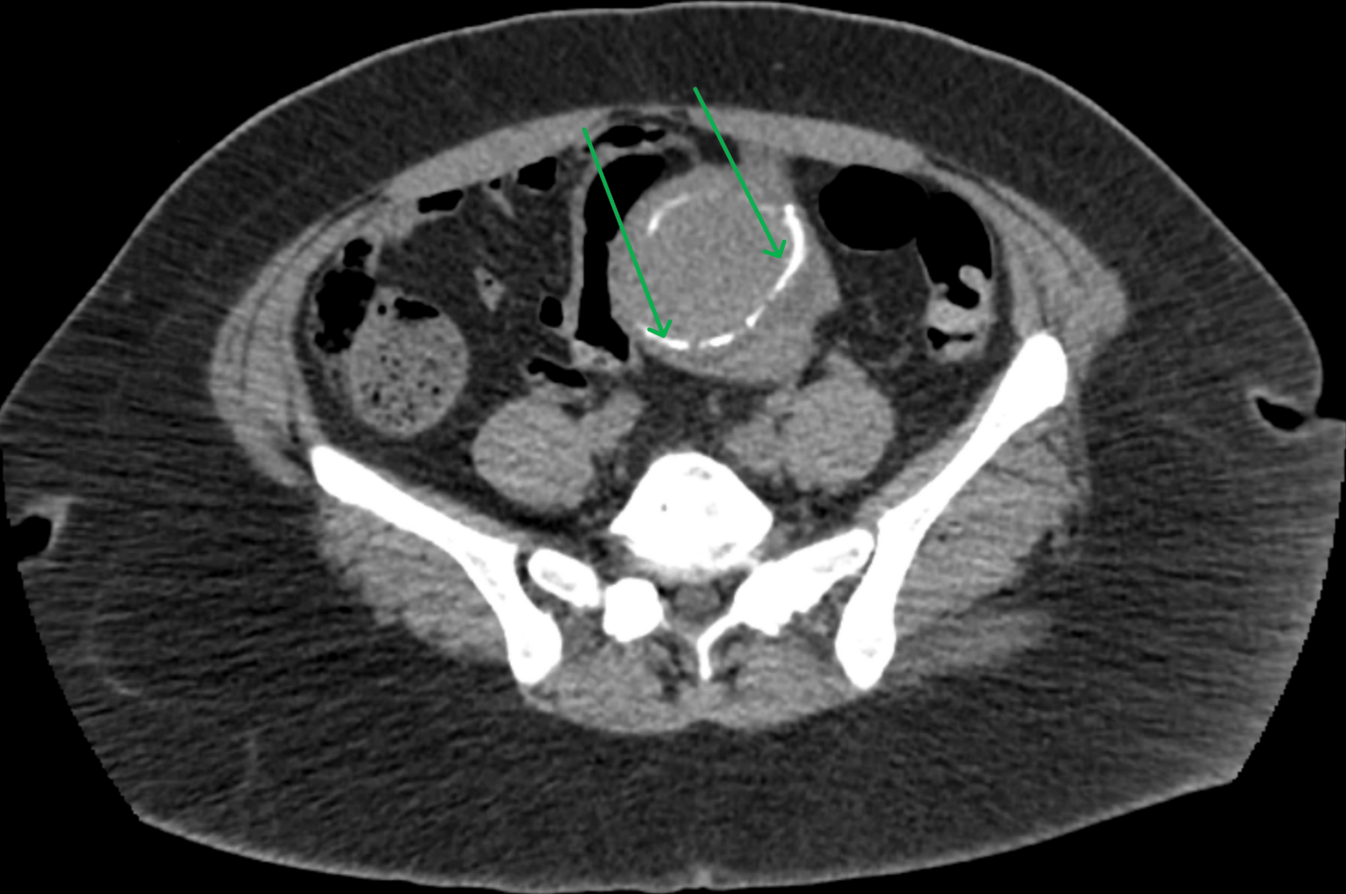
Abdomino-pelvic CT scan 2 Axial abdominopelvic CT scan at the level of the uterus. The image demonstrates a rim of calcified fibroid within the uterine wall, indicative of a degenerating leiomyoma. No evidence of bowel obstruction, perforation, or abscess.

On day 5 of hospitalization, a CT scan of the abdomen and pelvis (Figures 4, 5) was ordered to rule out an intra-abdominal etiology of the bacteremia. However, it was unrevealing and only showed evidence of uterine fibroids and a right ovarian cyst.

During hospitalization, the patient also reported headaches, which were evaluated by the Neurology team and determined to be migrainous in nature. She was treated with sumatriptan 50 mg for symptom relief. By hospital day 5, her condition began to improve, and she remained afebrile. She was completely asymptomatic by day 6. Repeat blood cultures obtained on day 5 were negative, and she was deemed clinically stable for discharge on day 7.

She received a week-long course of intravenous antibiotics during her hospital stay, followed by a 10-day course of oral levofloxacin upon discharge. She was also referred to Gynecology for outpatient follow-up regarding the uterine fibroid.

In the absence of an identifiable infectious source, the patient’s recent tattoo was considered the most plausible entry point for bacteremia. This case is particularly notable for its atypical presentation, including neurological symptoms and infection with a gram-negative organism not typically associated with tattoo-related complications.

## Discussion

Bacteremia following tattooing can present atypically, with neurological symptoms, no visible signs of infection at the tattoo site, and involvement of uncommon pathogens such as gram-negative bacteria. This case illustrates gram-negative bacteremia caused by *E. coli*, a pathogen not previously reported in tattoo-associated infections.

Localized infections after tattooing often present with redness, swelling, pain, or pus. For example, Elegino-Steffens et al. (2013) reported a case of a man who developed localized swelling that progressed to septic shock following traditional Samoan tattooing, with methicillin-resistant *Staphylococcus aureus *(MRSA) and Group A *Streptococcus* implicated as causative agents [10]. Poor antiseptic practices in traditional or unregulated tattooing often contribute to such infections [10]. Similarly, the CDC documented multiple MRSA outbreaks between 2004 and 2005, linked to non-sterile tattooing, with skin manifestations such as abscesses and folliculitis [11].

In contrast, systemic infections without local skin involvement are less common. For instance, Newell et al. (2022) described sepsis with infective endocarditis following tattooing [12], while Chalmers et al. (2010) reported xanthogranulomatous pyelonephritis in a similar context [13]. Both cases involved *S. aureus*. Unlike these reports, our patient presented with *E. coli* bacteremia, highlighting the variability in pathogen involvement and clinical presentation.

Gram-positive organisms such as *Staphylococcus* and *Streptococcus* species are the predominant pathogens in tattoo-associated infections [14-16]. However, gram-negative bacteria, such as *Pseudomonas aeruginosa* and *Haemophilus influenzae*, have also been implicated in rare cases [17], typically presenting with skin manifestations such as cellulitis or abscesses [16]. The involvement of *E. coli *in our case, without localized skin signs, adds a unique dimension to tattoo-related complications.

The patient underwent tattooing by a licensed professional, with no reported outbreaks among peers. However, potential lapses in infection control (e.g., hand hygiene, antisepsis) or improper aftercare may have contributed to her infection [12,18,19]. Additionally, her uncontrolled diabetes likely increased susceptibility, as seen in previous tattoo-related infections. Furthermore, prior studies have identified microbial contamination in unopened tattoo inks, suggesting multifactorial risks in tattoo-associated infections [19].

Gram-negative bacteremia, including cases caused by *E. coli*, is increasing in prevalence but remains largely understudied [20]. This trend has raised public health concerns and underscores the need for further research into its pathogenesis and management [20]. In fact, in 2005, *E. coli* surpassed *Staphylococcus aureus* as the most prevalent bloodstream pathogen [20].

While *E. coli* bacteremia is typically linked to UTIs or gastrointestinal sources [21], no such origin was identified in this case. The patient showed no clinical signs of UTI or pyelonephritis, and imaging revealed no infectious etiology. The recent tattoo application, therefore, emerged as the most likely source, suggesting an alternative entry route for E. coli into the bloodstream.

Risk factors for *E. coli *bacteremia include immunosuppression and underlying comorbidities [21,22], both of which were present in this patient. Her poorly controlled diabetes likely impaired immune function and wound healing, further increasing her susceptibility. Subtle breaches in infection control during the tattooing process may have facilitated bacterial entry, even in the absence of visible local signs.

Understanding the pathogenic mechanisms of *E. coli* in bacteremia is critical for early recognition and management. *E. coli*’s ability to cause bacteremia is not linked to a single virulence factor but to a combination of mechanisms that enhance immune evasion, adherence, and metabolic adaptability [20]. Capsule production protects against immune opsonization [23], while the pic gene modulates the immune response by reducing leukocyte activity, aiding bacterial survival in the bloodstream [24].

To determine the source of the bacteremia, extensive evaluations were conducted. Since *E. coli* bacteremia is commonly associated with UTIs or gastrointestinal translocation, these potential sources were thoroughly investigated. The patient had no signs of a UTI, and imaging did not show pyelonephritis or abscesses, suggesting that the bacteremia likely led to secondary seeding into the urine rather than originating from a urinary source.

Similarly, gastrointestinal translocation was considered, but the patient had no recent gastrointestinal symptoms such as diarrhea or abdominal pain, nor known risk factors for increased gut permeability. Given the lack of alternative sources, the recent tattoo application was considered the most likely point of entry for *E. coli* into the bloodstream.

Several factors support this association between tattoos and bacteremia. First, no other infectious sources were identified despite a thorough investigation. Second, breaches in skin integrity, even without visible local infection, can serve as a portal for bacterial entry, particularly in individuals with compromised immune function, such as those with poorly controlled diabetes. Lastly, previous reports have documented bacteremia following tattooing, primarily with gram-positive organisms. This case underscores the need to consider gram-negative pathogens as potential culprits and emphasizes the importance of strict infection control practices in tattoo procedures.

The clinical presentation of bacteremia and sepsis can vary based on patient factors, the pathogen, and the infection source. Sepsis, defined as a dysregulated immune response to infection leading to life-threatening organ dysfunction, can affect multiple systems, including the respiratory, hepatic, cardiovascular, renal, coagulation, and central nervous systems [20]. Neurological complications of *E. col*i bacteremia in adults include confusion, altered mental status, and severe septic encephalopathy [25,26]. Sepsis-associated encephalopathy, characterized by cognitive impairment, delirium, and altered consciousness, results from systemic inflammation, blood-brain barrier dysfunction, and cerebral hypoperfusion [27]. A study during the 2011 outbreak of Shiga toxin-producing *E. coli *O104:H4 infection reported that 48% of patients with complicated infections developed neurological symptoms, including cognitive impairment, aphasia, and seizures [26]. These complications underscore the importance of early identification and prompt management to reduce the risk of long-term morbidity.

Limitations

Single Case Report

As this is a single case report, the findings may not be generalizable to the broader population. Larger cohort studies are needed to better understand the relationship between tattooing and gram-negative bacteremia, particularly with uncommon pathogens like E. coli.

Uncertainty in Infection Control Practices

While there was no evidence of lapses in infection control during the tattoo procedure, it remains possible that subtle breaches in hygiene or antisepsis practices contributed to the infection, but these could not be directly observed or documented.

Absence of Long-Term Follow-up

The article does not provide long-term follow-up data to assess potential long-term sequelae or the recurrence of infection. Further studies would benefit from tracking patients over a longer period to understand the full scope of outcomes after tattoo-related bacteremia.

## Conclusions

This case highlights the rare occurrence of gram-negative bacteremia, specifically caused by *E. coli*, following tattooing, an uncommon pathogen in tattoo-related infections. Despite the absence of local infection at the tattoo site, the patient presented with systemic symptoms, including neurological manifestations, emphasizing the need to consider atypical clinical presentations in such cases. While no alternative infectious sources were identified, the patient’s risk factors, including poorly controlled diabetes, suggest a possible association between the tattoo and bacteremia.

This case underscores the importance of infection control during tattoo procedures, particularly in immunocompromised individuals. Clinicians should remain vigilant for bacteremia in patients with unexplained systemic symptoms, even in the absence of visible local infection. Further research is needed to better understand tattoo-related bacteremia and to develop clear diagnostic and therapeutic guidelines for such rare but serious complications.
